# Aneuploid Circulating Tumor-Derived Endothelial Cell (CTEC): A Novel Versatile Player in Tumor Neovascularization and Cancer Metastasis

**DOI:** 10.3390/cells9061539

**Published:** 2020-06-24

**Authors:** Peter Ping Lin

**Affiliations:** Cytelligen, San Diego, CA 92121, USA; plin@cytelligen.com; Tel.: +1-(858)-336-5788; Fax: +1-(858)-509-9209

**Keywords:** aneuploid CTC, aneuploid CTEC, cellular circulating tumor biomarker, endothelialization of cancer cells, cancerization of stromal endothelial cells, EMT, EndoMT, transdifferentiation, cell fusion, hypoxia, iFISH

## Abstract

Hematogenous and lymphogenous cancer metastases are significantly impacted by tumor neovascularization, which predominantly consists of blood vessel-relevant angiogenesis, vasculogenesis, vasculogenic mimicry, and lymphatic vessel-related lymphangiogenesis. Among the endothelial cells that make up the lining of tumor vasculature, a majority of them are tumor-derived endothelial cells (TECs), exhibiting cytogenetic abnormalities of aneuploid chromosomes. Aneuploid TECs are generated from “cancerization of stromal endothelial cells” and “endothelialization of carcinoma cells” in the hypoxic tumor microenvironment. Both processes crucially engage the hypoxia-triggered epithelial-to-mesenchymal transition (EMT) and endothelial-to-mesenchymal transition (EndoMT). Compared to the cancerization process, endothelialization of cancer cells, which comprises the fusion of tumor cells with endothelial cells and transdifferentiation of cancer cells into TECs, is the dominant pathway. Tumor-derived endothelial cells, possessing the dual properties of cancerous malignancy and endothelial vascularization ability, are thus the endothelialized cancer cells. Circulating tumor-derived endothelial cells (CTECs) are TECs shed into the peripheral circulation. Aneuploid CD31^+^ CTECs, together with their counterpart CD31^-^ circulating tumor cells (CTCs), constitute a unique pair of cellular circulating tumor biomarkers. This review discusses a proposed cascaded framework that focuses on the origins of TECs and CTECs in the hypoxic tumor microenvironment and their clinical implications for tumorigenesis, neovascularization, disease progression, and cancer metastasis. Aneuploid CTECs, harboring hybridized properties of malignancy, vascularization and motility, may serve as a unique target for developing a novel metastasis blockade cancer therapy.

## 1. Introduction 

Aberrant stromal cells, sustained neovascularization, dysfunctional angiogenic vasculature, and infiltrated immune cells contribute to a tumor microenvironment (TME) suitable for tumor growth and cancer metastasis [1]. Tumor neovasculature in the TME is principally comprised of blood vessels and lymphatic vessels. Hematogenous and lymphogenous cancer metastases are significantly impacted by tumor neovascularization [2], of which the latter is predominantly comprised of endothelium-dependent angiogenesis, vasculogenesis, lymphangiogenesis, and endothelium-independent vasculogenic mimicry (VM). Both epithelial-to-mesenchymal transition (EMT) and endothelial-to-mesenchymal transition (EndoMT) are involved in the neovascularization process [3,4]. The cascades of cancer metastasis and tumor neovascularization are induced and regulated by hypoxia [5,6], which facilitates the release of angiogenic factors (such as VEGF, PDGF, TNF-α, and IL-8) that promote the proliferation of quiescent endothelial cells (ECs). 

Among various cellular components identified in the TME, ECs that make up the lining of the tumor vasculature are at the center of diverse processes involved in the pathogenesis of malignant neoplasms [4]. A majority of ECs in the tumor vasculature are tumor-derived ECs (TECs) exhibiting cytogenetic abnormalities of aneuploid chromosomes [7,8]. These aneuploid TECs [9,10], which could be directly derived from malignant cancer cells, harbor mixed properties of both endothelial vascularization ability and cancerous malignancy in the TME. Morphologically abnormal tumor vasculature, contributed by the TECs, possesses loosened junctions between ECs. This leads to an increase in vascular permeability and transendothelial intravasation as well as extravasation during tumor metastasis. Following their shedding into peripheral blood, TECs turn into circulating tumor-derived endothelial cells (CTECs) [11]. Some CTECs in different types of carcinoma patients were found to express a variety of tumor and mesenchymal markers, such as HER2, PD-L1, EpCAM, CD44v6 and Vimentin [11,12,13]. Aneuploid CD31^+^ CTECs and their counterpart CD31^–^ CTCs compose a unique pair of cellular circulating tumor biomarkers in cancer patients [12,14].

Several proposed mechanisms and detailed paradigms have segmentally provided depictions accounting for the roles of TECs in tumor vascularization, progression, and metastasis. However, the underlying mechanisms regarding how TECs and CTECs arise during tumorigenesis and progression, and how those cells acquire an aneuploidy characteristic, a hallmark of malignancy, are yet to be more clearly and systematically elucidated. In the current review, the origin and clinical significance of CTECs are consecutively discussed along the axis of “formation mechanisms”, “hypoxia regulation”, and “therapeutic target potential” to provide new insights into CTECs’ impact on tumorigenesis, neovascularization, disease progression, and cancer metastasis. 

## 2. Tumor Microenvironment

The TME is a complex, dynamic system comprised of tangled interactions among cancer cells and their surrounding cells. Aside from non-cellular components, such as extracellular matrix and soluble factors (cytokines), the cellular composition of the TME consists of highly tumorigenic undifferentiated cancer stem cells (CSCs) [15] and their differentiated progeny tumor cells that possess intrinsic or induced plasticity [16]. In addition, a variety of stromal cells that are able to foster both tumor growth and dissemination are also found in the TME. These cells are composed of ECs, cancer-associated fibroblasts (CAFs), pericytes, adipocytes, and immune cells (lymphocytes, dendritic cells, monocytes as well as macrophages). Tumor-infiltrating lymphocytes, containing CD3^+^/CD4^+^ and CD3^+^/CD8^+^ T cells, are the major cellular components of lymphocytes in the TME [17]. These infiltrated T cells are recognized as one of the hallmarks of cancer ever since the linkage between chronic inflammation and tumorigenesis was first proposed by Rudolf Virchow more than a century ago [18]. Active bidirectional communication between neoplastic cells and their associated stromal cells significantly impacts cancer initiation and development [1]. For instance, in a cellular microenvironment, senescent stromal fibroblasts acquire a senescence-associated secretory phenotype (SASP) that turns senescent fibroblasts into proinflammatory cells and subsequently promote tumor progression [19]. 

The TME provides support for tumor vasculature, which is crucial for maintaining and promoting tumor growth. Tumor vascularization requires an interaction among multiple types of malignant and non-tumorigenic cells in the TME, including ECs that form tight adhesions so as to maintain the structural integrity of blood and lymphatic vessels, pericytes that provide vessels’ coverage and dictate vessel maturity, as well as bone marrow (BM)-derived precursor cells, such as epithelial progenitor cells (EPCs), whose differentiation is mainly regulated by hypoxia [20]. All additional accessory cells including mesenchymal stromal cells (MSCs) [21], tumor-associated macrophages, and cancer-associated fibroblasts (CAFs) participate in tumor progression and vascularization. Particularly, in the TME, CAFs are the most abundant stromal cells and engage in the remodeling of peritumoral stroma, a prerequisite to cancer cell invasion and expansion [4]. Moreover, CAFs interact with cancer cells and form a myofibroblastic microenvironment that fosters tumor growth and survival and supports malignancy, thereby promoting disease progression in cancer patients [22,23].

## 3. Tumor Neovascularization and Cancer Metastasis 

### 3.1. Tumor Neovascularization

Tumorigenesis, tumor growth, and metastasis are dependent on the development of surrounding functional vasculature. Two major endothelium-based strategies have been proposed to account for how carcinomas develop neovasculature in the TME: (a) angiogenesis and (b) vasculogenesis. Endothelium-independent vasculogenic mimicry (VM) is an additional approach that supplies blood necessary for neoplasms.

Angiogenesis is the process of forming new capillary blood vessels and lymphatic vessels. Two different types of angiogenesis have been discovered: sprouting angiogenesis, which is responsible for vascular growth and the alternative nonsprouting intussusceptive angiogenesis, which participates in both growth and remodeling of vascular beds of tumors *via* splitting pre-existing vessels into daughter vessels [24]. Sprouting angiogenesis is the primary process which accounts for tumor neovascularization via sprouting, migration, growth, and proliferation of the quiescent, matured, differentiated ECs in nearby pre-existing blood vessels to generate new blood vessels. Matured ECs line the interior wall of the newly formed branches of vessels or entangle with carcinoma cells in the tumor blood vessel wall to form a mosaic vasculature [25]. Angiogenesis, promoted by CSCs through stimulation of the vascular endothelial growth factor (VEGF), is a characteristic trait of carcinomas [26] and is necessary for all invasive cancers’ initiation, growth, metastasis, and control of malignant tumor progression [27]. 

Vasculogenesis is a vascularization process centered on recruiting BM-derived precursor cells, including EPCs and pericyte progenitor cells in circulation, that subsequently differentiate into ECs, followed by the *de novo* formation of vasculature with those differentiated ECs in the TME [28,29]. The entire process is regulated by hypoxia [30], carcinomas (such as breast cancer) [31], chemokines, cytokines, angiogenic factors [32], and Notch [28,33]. Angiogenesis and vasculogenesis are the two primary endothelium-based strategies by which tumors develop neovasculature [34]. Angiogenesis is the dominant pathway during neovascularization, while vasculogenesis is the prime backup pathway in practice when local angiogenesis is therapeutically abrogated [28].

Unlike the endothelium-dependent vasculature, vasculogenic mimicry (VM) provides an endothelium-independent approach of supplying nutrients to neoplasms [32,35]. Some malignant neoplastic cells with high plasticity in VM revert to dedifferentiate into endothelial-like CSCs [36]. These CSCs converge in vasculogenic-like channels that connect to angiogenesis- and vasculogenesis-derived host blood vessels. VM channels are independent of angiogenesis and do not have an EC lining. Malignant carcinomas containing VM include breast, gastric, ovarian, prostate, renal cell, and hepatocellular (HCC) carcinomas and most sarcomas [35,37]. Although not as frequent as angiogenesis and vasculogenesis, VM channels directly expose tumor cells to blood flow, thus resulting in increased cancer metastasis potential and poor prognosis in most patients with various types of carcinomas [35,38]. 

Along with the aforementioned diverse types of tumor vascularization, vessel co-option serves as another means for tumors to obtain blood. In vascular co-option, instead of depending on neovasculature, cancer cells hijack the pre-existing vasculature in the host organ and acquire essential supplies. Moreover, these carcinoma cells, often referred to as the non-angiogenic metastatic neoplastic cells, can migrate along the existing vessels to distant organs [39]. 

### 3.2. Hematogenous and Lymphogenous Cancer Metastases

Cancer metastasis consists of two fundamental pathways: hematogenous metastasis via blood vessels (post-neovascularization) and lymphogenous metastasis via the lymphatic system (post-lymphangiogenesis). Neoplastic cells from primary lesions may directly intravasate into the blood and start their journey of hematogenous distant metastasis. On the other hand, cancer cells in the TME may initiate their lymphogenous metastasis process via penetrating into lymphatic vessels and disseminating to sentinel then distant lymph nodes through lymph flow. Cancer cells in the nodes subsequently enter the thoracic duct and subclavian vein, and ultimately metastasize to the distant target organ. [40,41,42]

In contrast to the blood vessels that deliver oxygen and nutrients to the tumor, the lymphatic system, with blind-ended capillaries in tissues and an open layout toward the blood, only absorbs extravasated fluids, lipids, and immune cells in its lymph in a unidirectional manner flowing from tissue to peripheral blood. Lymphatic vessels are comprised of three types of ECs, including lymphatic ECs originated from pre-existing lymphatic vessels, blood vessel ECs, and bone marrow-derived progenitor cells which will either differentiate or transdifferentiate into lymphatic ECs. These three types of ECs constitute the newly formed lymphatic vessels in the neo-lymphangiogenesis process induced by the primary tumor [41]. An active cross-talk between lymphatic and blood vessel ECs in the TME has been proposed to impact cancer cells’ selection of the hematogenous or lymphogenous metastasis pathway [40,41]. 

## 4. Mechanism of Tumor-Derived EC and CTEC Formation: Endothelialization of Malignant Cancer Cells and Cancerization of Stromal Cells

Similar to cancer cells shed into peripheral blood (CTCs) and BM (known as disseminated tumor cells, DTCs) [43,44], aneuploid TECs are also detected in peripheral circulation as CTECs [11] or in ascites, malignant pleural effusion, cerebrospinal fluid, and BM as disseminated tumor-derived ECs (DTECs). From previous research, light has been shed on the clinical relevance of TECs in cancer metastasis and tumor progression [9,45]. However, the origin of TECs and the molecular mechanisms regarding how TECs harbor the dual properties of malignancy and vasculogenic ability remains to be concisely elucidated. 

The origin of TECs is composed of two distinct processes: cancerization of stromal cells and the predominant endothelialization of malignant tumor cells. A vast amount of evidence indicates that epithelial carcinoma lesions do not progress into malignancy even if they share the genetic variations identified in invasive and metastatic cancers. Changes in surrounding tumor stroma, such as CAFs, have been recognized to play a primary role in both tumorigenesis and disease progression. These alternation processes are termed as “cancerization” or “field cancerization” [46]. Accordingly, distinct epigenetic molecular variations observed with methylation-specific digital karyotyping on stromal cells have been confirmed [47]. Like karyotypic alternations in stromal CAFs during cancerization, which co-evolves initiation and disease progression of epithelial solid tumors [22,23], stromal ECs are expected to follow a similar cancerization process in their conversion into aneuploid TECs. 

In regard to cancer cell endothelialization, visual evidence of “cellular endothelialization” of tumor cells’ participation in lung cancer metastasis was reported [48]. Phenotypic and karyotypic “molecular endothelialization” is proposed to enable the transdifferentiation of neoplastic cells into CD31^+^ aneuploid TECs. Compared to stromal EC cancerization, endothelialization of malignant cancer cells, comprised of transdifferentiation and heterotypic cell fusion, may constitute the primary pathway for the formation of TECs.

### 4.1. Transdifferentiation 

During the processes of endothelialization of cancers and cancerization of ECs, both tumor cells and stromal cells can transdifferentiate into TECs. In cancer patients, tumors contain highly tumorigenic undifferentiated CSCs that have both multi-lineage differentiation and transdifferentiation potentials [16]. CSCs could develop into phenotypically differentiated tumor cells (non-CSCs) via the produced poorly differentiated tumor progenitor cells or transdifferentiate into cell lineages other than the original lineage from which the tumor arose, thereby promoting tumor initiation, neovascularization, proliferation, and metastasis [16]. Transdifferentiation of tumor cells into ECs refers to the process by which carcinoma cells adopt a more mesenchymal stem-like phenotype following EMT (i.e., dedifferentiation process). The produced mesenchymal intermediate CSCs subsequently transdifferentiate into TECs/ECs [16,36]. The transdifferentiation of cancer cell-derived CSCs into TECs/ECs was observed in a variety of carcinomas, including epithelial cancers of the breast [49], ovary [50], and renal cell [51], as well as other lineages of tumors, such as glioblastoma [33]. CSCs, therefore, are the central nodes of transdifferentiation. Among the transdifferentiated TECs in carcinoma patients, some express TMs as either shown in this review or demonstrated previously by us [11,12,13]. 

Regarding the transdifferentiation process in the cancerization of stromal cells, the central component (i.e., MSCs) is contributed by dedifferentiation of ECs and other stromal cells through EndoMT [4,52,53] in addition to derivation from embryonic stem cells (ESCs) and induced pluripotent stem cells (iPSCs) in BM. The formation of MSCs through EndoMT provides new insights into the mechanisms involved in the establishment of MSCs [53]. The obtained undifferentiated MSCs subsequently differentiate into mesodermal derivatives including adipocytes, osteocytes, and chondrocytes [54]. Moreover, EC-derived MSCs are also able to differentiate into the same lineage of TECs/ECs, both in vivo and in vitro [54,55]. The process of differentiation from EC-derived MSCs or transdifferentiation from other stromal cell-derived MSCs into ECs/TECs is termed as the “stromal cell–MSC–EC transition cycle” [53], which, together with the senescence of cells discussed below, may account for the infrequent existence of abnormal aneuploid ECs without TM expression in some healthy subjects [11]. These individual, occasionally existent, abnormal, null ECs in peripheral blood, such as abnormal circulating endothelial cells (CECs), could be homeostatically depleted by the immune scavengers of the host defense system [56].

### 4.2. Heterotypic Cell Fusion

Cell fusion is a fundamental process in morphogenesis, tissue regeneration, and homeostasis. In contrast to homotypic fusion, heterotypic fusion of diverse lineages of cells yields multinucleated (heterokaryons) or mononucleated hybrid cells (synkaryons) [57]. The resultant cell hybrids are important for the pathogenesis of diseases that lead to the initiation and progression of neoplasms [58]. In fact, many neoplastic cells are fusogenic, generating enhanced malignant multinuclear hybrid cancer cells in which nucleic DNA undergoes asynchronous DNA synthesis or mitosis [59]. Through fusion of different lineages of cells (such as cancer cells with ECs) in the TME, fusogenic tumor cells reprogram their genomes in the resultant hybrids and exhibit novel properties, including increased tumor cell heterogeneity and systemic metastasis, via the assistance of cancer cells to precisely target their respective organs for the metastatic outgrowth [60,61]. 

Aneuploidy has been recognized as one of the hallmarks of malignant neoplastic cells [62,63,64]. Of particular interest is that homotypic or heterotypic cell fusion may partially account for the aneuploidy of chromosome(s) [59,63], leading to genomic instability [65]. It has been proposed that cancer cells are prone to cell fusion, resulting in multiploid hybrid cells with aberrant chromosome segregation, followed by progression into aneuploidy. Cell fusion accelerates genetic and epigenetic variability, giving rise to cancer cells’ new hybridized properties of different lineages, such as the mixed phenotype and/or karyotype of parental cells [66]. Besides cell fusion, some senescent cells, such as human umbilical vein ECs (HUVECs), were found to be arrested in the G1 phase, resulting in polyploidized tetraploid HUVECs with 4N DNA content, which further triggered self-cleaning-relevant apoptosis for homeostasis [67]. Nonetheless, these apoptotic senescent tetraploid cells, if shed in circulation, are not expected to have specific clinical relevance in cancer patients. 

Cell fusion provides the required metastatic phenotypes for neoplastic cells [61]. Primary malignant human cancer cells are prone to spontaneously fuse with a variety of cells, including ECs [61,68], to create fusogenic hybrids possessing metastatic properties and exhibiting dual phenotypic and karyotypic characteristics from each parental cell [61]. Communication between ECs and neoplastic cells is crucial for tumor angiogenesis, intravasation, and metastatic dissemination [69]. Heterotypic cell hybridization, facilitated by apoptosis via the phosphatidylserine receptor-associated PtdSer-BAI1 signaling pathway [70], involves an interaction between the fusogenic retroviral envelope protein “syncytin” (the ligand on cancer cells) and the syncytin receptor (alanine/serine/cysteine transporter-2, ASCT-2) expressed on ECs [71]. Such a fusogenic interaction is significantly facilitated in a TNF-α-induced inflammatory environment through the activation of vascular cell adhesion molecule-1 (VCAM-1) and ASCT-2 on ECs [72]. This promotes tumor angiogenesis and transendothelial intravasation. Quantified cancer cell–EC fusogenic hybrids in pancreatic cancer patients’ peripheral blood were found to proportionally correlate with malignant disease stages spanning from early to late stages, and were suitable for predicting patients’ overall survival [73]. Aside from fusion with ECs, cancer cells may also fuse with MSCs to gain EMT properties that will assist specific organ-targeted cancer metastasis [61,74].

### 4.3. Schematic Depiction of Transdifferentiation and Cell Fusion 

**Figure 1:**
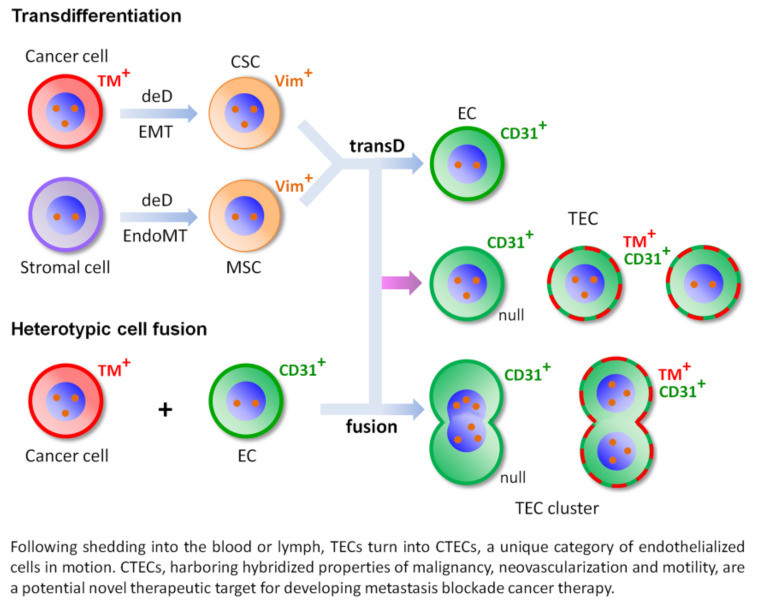
Schematic diagram of transdifferentiation and heterotypic cell fusion. Aneuploid tumor-derived endothelial cells (TECs) and circulating tumor-derived endothelial cells (CTECs) in carcinoma patients are primarily contributed by transdifferentiation and heterotypic cell fusion. (**A**) Transdifferentiation (transD). Differentiated aneuploid cancer cells and stromal cells dedifferentiate (deD) into Vimentin^+^ (Vim^+^) mesenchymal cancer stem cells (CSCs) and mesenchymal stromal cells (MSCs) via epithelial-to-mesenchymal transition (EMT) and endothelial-to-mesenchymal transition (EndoMT), respectively. The resultant undifferentiated CSCs and MSCs may transD into individual normal diploid CD31^+^ endothelial cells (ECs, top blue arrow) and/or aneuploid TECs (middle pink arrow) having either a positive expression of tumor markers (TMs) (red/green dash ring, diploid or aneuploid) or not (null, aneuploid, green). Among MSCs, some EC-derived MSCs may differentiate into the same endothelial lineage of TECs/ECs. (**B**) Heterotypic cell fusion. An aneuploid cancer cell fuses with an EC to generate either an individual, mononuclear synkaryon of aneuploid TEC (pink arrow) or a multinuclear heterokaryon in a cluster (microemboli, bottom blue arrow). The fusogenic hybrids may or may not (null) have a positive expression of TMs.

As depicted in Figure 1, heterotypic cell fusion and transdifferentiation of cancer cells and stromal cells into TECs/ECs, respectively, via CSCs and MSCs, participate in aneuploid TEC and CTEC formation in cancer patients. Among stromal cells, some stromal EC-derived MSCs may differentiate into the same endothelium lineage of TECs/ECs. Compared to transdifferentiation, cell fusion principally accounts for the observed cell identity changes [75]. Among individual non-hematologic CTECs [12,13] and their clusters [11,12] obtained from either transdifferentiation or cell fusion, those harboring a mixed phenotype of both endothelial lineage and various tumor markers (TMs) are either aneuploid or infrequently diploid.

**Figure 2:**
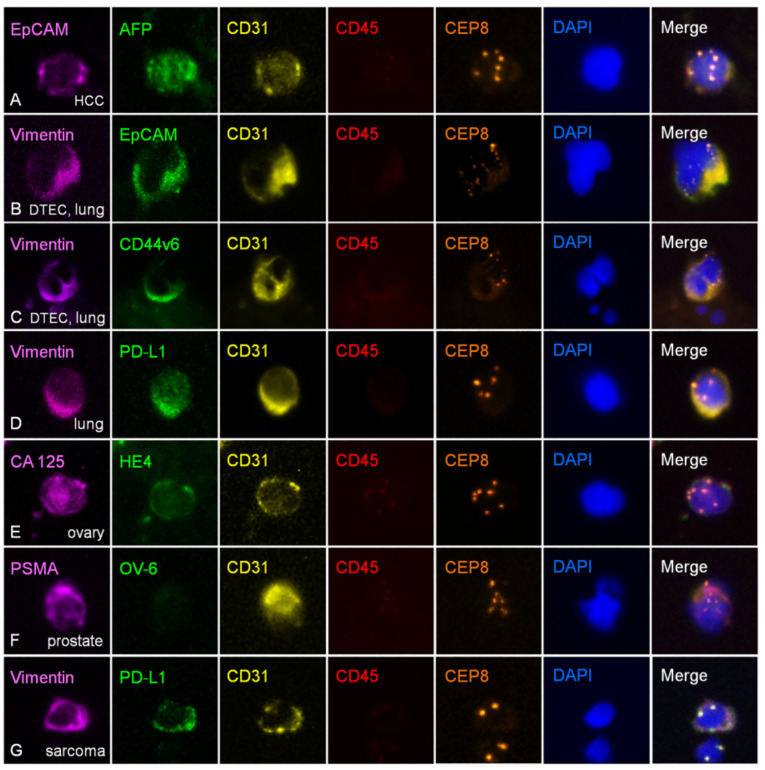
In situ phenotypic and karyotypic characterization of aneuploid CTECs and disseminated tumor-derived ECs (DTECs) detected in varieties of cancer patients by the integrated immunostaining-fluorescence *in situ* hybridization (iFISH). Aneuploid TECs in peripheral blood (CTECs) and bone marrow (BM; DTECs) of a variety of carcinoma patients were enriched by subtraction enrichment (SE), followed by 6-channel iFISH to simultaneously co-characterize aneuploidy of chromosome 8 and TM expression in CD31^+^/CD45^–^ CTECs and DTECs. (**A**) An aneuploid hepatocellular carcinoma (HCC) CTEC enriched from blood had plasma membrane staining of the stemness marker EpCAM and cytoplasmic vesicular staining of secretory α-fetoprotein (AFP). (**B**) A multinuclear mesenchymal (Vimentin^+^) and EpCAM^+^ DTEC fusion cluster enriched from bone marrow in a non-small cell lung cancer (NSCLC) patient (DTEC microemboli). The cell cluster in BM had dual phenotypes of both epithelium and mesenchyme. (**C**) A multinuclear mesenchymal (Vimentin^+^) and stemness marker CD44v6^+^ DTEC fusogenic microembolus in BM of a lung cancer patient. The cell cluster in BM was composed of disseminated tumor-derived endothelial stem cells (DTESCs) with a mesenchymal phenotype. (**D**) An aneuploid mesenchymal (Vimentin^+^) NSCLC CTEC revealed a granule-like staining of PD-L1, suggesting that PD-L1 may localize in the secretory granules. (**E**) An aneuploid ovarian cancer CTEC showed a positive staining of cytoplasmic vesicle-like CA 125 and human epididymis protein 4 (HE4). (**F**) An aneuploid prostate cancer CTEC had a positive plasma membrane and cytoplasmic staining of the prostate-specific membrane antigen (PSMA), but negative for the oval cell marker OV-6 staining. (**G**) A diploid mesenchymal (Vimentin^+^) and PD-L1^+^ sarcoma CTEC. Fluorescence dyes conjugated to diverse antibodies (Cytelligen, USA): Alexa Fluor 488 (green), AF594 (red), Cyanine 5 (yellow), and Cy7 (purple).

As discussed above, although some non-clustered, mononuclear, polyploid, abnormal null CECs derived from stromal cell transdifferentiation or cellular senescence were occasionally detected in healthy subjects [11], multinuclear fusogenic aneuploid EC clusters were observed only in the diagnosed cancer patients’ peripheral blood (termed as CTEC microemboli) [11,12] or bone marrow (DTEC microemboli shown in Figure 2), not in healthy donors. It is interesting to point out that in cancer patients, besides those expressing TMs, many aneuploid CD31^+^ CTECs have no TM expression, namely the null CTECs. An investigation of how TM expression in CTECs is regulated and a comparison of the clinical relevance of null and TM-positive CTECs remain to be further expanded [12].

### 4.4. Comprehensive Phenotypic and Karyotypic Characterization of TM^+^ CTECs and DTECs in Varieties of Cancer Patients 

Despite the existence of null CTECs in carcinoma patients, some CD31^+^/CD45^–^ CTECs expressing various TMs, such as HER2, PD-L1, and EpCAM, were detected by the integrated strategy of “subtraction enrichment-immunostaining-fluorescence *in situ* hybridization (SE-iFISH)” (Cytelligen, USA) [11,14,76] in varieties of cancer patients [11,12,13]. Additional TM^+^ mononuclear aneuploid CTECs, DTECs, and their multinuclear heterokaryon clusters in cancer patients are revealed in Figure 2. Given that TECs/ECs are secretory cells [77], CTECs, revealed in Figure 2, were accordingly found to have intracellular α-fetoprotein (AFP), CA 125, and secreted PD-L1 [78] in cytoplasmic secretory vesicles or regulated secretion granules (RSGs) along the constitutive or regulated secretory pathway, as exhibited by the multidestination protein, Calnuc [79,80].

Apart from CTECs and DTECs, diverse subtypes of aneuploid CTCs and DTCs expressing various TMs detected by the same SE-iFISH strategy in numerous cancer patients [81,82,83] and metastatic PDX tumor mouse models [84] were also previously demonstrated. Moreover, *in situ* co-detection and co-investigation of the synergetic clinical significance of aneuploid CTECs and CTCs have been recently reported [12].

## 5. Hypoxia Induces Cancer Metastasis and Tumor Neovascularization 

Hypoxia, a reduced supply of oxygen, is a common phenomenon in malignant carcinomas that leads to neoplastic and endothelial cells’ acquisition of EMT and EndoMT phenotypic plasticity. This results in abnormal vascularity, dysfunctional vascularization, enhanced permeability, increased cell motility, and increased cancer metastasis [5,6]. Hypoxia-induced EMT and EndoMT are the central hub of cancer metastasis and tumor neovascularization. Although a number of signaling pathways are activated by hypoxia in carcinoma cells, the hypoxia-inducible factor (HIF) pathway is the most distinctive due to its regulation of EMT and EndoMT. Apart from non-hypoxia induction in response to cytokines and growth factors (such as transforming growth factor-β, TGF-β) mediated by the NFκB, MAPK, and PI3K/AKT/mTOR pathways [85,86], the HIF pathway is primarily activated in hypoxic environments, during which the non-hydroxylated HIF-1α subunit translocates into the nucleus and dimerizes with HIF-1β. This is followed by subsequent DNA binding and initiation of relevant downstream gene transcription [87] that promotes neovascularization and metastasis cascades [5,6].

### 5.1. Hypoxia Induces EMT and EndoMT

Epithelial-to-mesenchymal transition (EMT) prompts epithelial cancer cells to acquire the mesenchyme phenotypes and turn those cells into CSCs. Similarly, stromal cells or tumor-associated stromal cells [88] (ECs, pericytes, etc.) are converted into mesenchymal MSCs through endothelial-to-mesenchymal transition (EndoMT). CSCs and MSCs both exhibit noteworthy significance in cancer metastasis and tumor neovascularization.

#### 5.1.1. Hypoxia-Induced EMT: An Essential Process in Cancer Metastasis and CSC Formation 

EMT, an evolutionarily conserved developmental program, plays a vital role in promoting cancer metastasis by enhancing cancer cell dissemination and motility [89,90]. The activation of EMT allows for the regulation of several molecules including E-cadherin and EpCAM in tumor cells and causes these cells to lose cell junctions, gain mesenchymal properties, followed by dissociation from the original tumor mass and the initiation of metastasis cascade [90,91,92]. 

The most representative epithelial marker, EpCAM (CD326), known as the epithelial cell adhesion molecule, is a transmembrane glycoprotein that mediates Ca^2+^-independent homotypic cell–cell adhesion in epithelia. In addition to being located on the plasma membrane, full-length EpCAM and its cleaved intracellular domain EpICD were also found in the lysosome and nuclei, respectively [93]. The nuclear EpICD is active in nuclear signal transduction, ensuring EpCAM’s ability to function in cell proliferation [93,94]. EpCAM is a key molecule that engages in cancer cell signaling, differentiation, proliferation, migration, progression, and invasion [93,94,95,96,97]. High expression of EpCAM was reported on epithelial cancer cells in primary and metastatic lesions, as well as on some undifferentiated CSCs of major carcinoma entities [98]. However, low expression was observed on differentiated CTCs and DTCs [44,82,99]. Dynamic changes in the epithelial and mesenchymal composition of CTCs were recently reported [99,100]. EpCAM^+^ and Vimentin^+^ aneuploid CTCs and CTECs, produced by the transdifferentiation of CSCs or heterotypic cell fusion of cancer cells with ECs, were detected in cancer patients with a variety of epithelial carcinomas [11,12,13,101]. EpCAM^+^ neoplastic cells showed diminished sensitivity toward growth factor deprivation and increased tumorigenicity [44]. Poor prognostic values of EpCAM^+^ tumor cells or CTCs in cancer patients were described elsewhere [95,102,103]. We identified that, among CTCs and DTCs from various entities, the unique population of EpCAM^+^ CTCs and DTCs with cytogenetic abnormalities of aneuploid chromosome 8 as the particular subtype accounting for the systemic metastasis in breast cancer patients [82] and the metastatic recurrence in postsurgical hepatocellular carcinoma patients [83]. 

In addition to promoting cancer metastasis, it has been recognized that EMT also participates in the process of CSC formation. CSCs are a small population of undifferentiated carcinoma cells that exhibit stem cell-like features of self-renewal and the ability to seed new tumors. During EMT, tumor cells dedifferentiate into undifferentiated CSCs. The undifferentiated CSCs have tumor-initiating properties, which in turn cooperate with EMT and are eventually converted into CTCs in blood and DTCs in the BM [43,44,104,105,106]. In the hypoxic TME, HIF-1α triggers the EMT program in epithelial cancer cells [107]. EMT is activated by a core set of EMT-inducing transcription factors (EMT-TFs), such as Twist1, Snail, Slug, ZEB1, and ZEB2 [108], which results in the carcinoma cells’ acquisition of stemness properties, thus turning them into CSCs exhibiting a mesenchymal phenotype [36,109,110]. Undifferentiated mesenchymal CSCs possess differentiation and transdifferentiation potentials [16]. In the TME, CSCs could transdifferentiate into cells (such as TECs) that differ from their own original lineage along the axis of Twist1-Notch-KLF4 [7] or differentiate into their original lineage of tumor cell progeny, of which the latter differentiation process is bidirectional, meaning that the differentiated tumor cells can conversely dedifferentiate into CSCs. The two-way conversion between differentiation and dedifferentiation is accelerated by hypoxia in the TME [16]. HIF, the key regulator of differentiation, dedifferentiation, and transdifferentiation of CSCs [109], helps maintain the plasticity in CSCs and their progeny, thereby contributing to neoplastic cell and CSC heterogeneity [16]. Several CSC phenotypes may co-exist with different types of carcinomas, each possibly possessing their own distinctive therapeutic susceptibility and metastatic potential. For instance, EMT-derived CSCs expressing mesenchymal phenotype take part in the initiation of cancer metastasis [111], and CSCs as cancer initiating cells (CICs) expressing the stemness phenotype CD44v6 (Figure 2) in varieties of carcinomas were found to have an increased metastatic potential [112,113]. In addition, the heterogeneity of CSCs is believed to contribute to guiding the metastatic CSCs to the specific target organ(s) during tumor spread [111].

#### 5.1.2. Hypoxia-Induced EndoMT: A Critical Process in MSC and CAF Formation and Vasculature Remodeling

EndoMT [4], or EndMT, is a complex process of endothelial-to-mesenchymal transition, which is categorized as a specialized version of EMT. During the “stromal cell–MSC–EC transition cycle” [53], EndoMT, sharing the common signaling molecules and pathways of EMT (such as Notch, Twist1, and Wnt/β-catenin), promotes the dedifferentiation of stromal cells to give rise to undifferentiated EC-derived [53] or other stromal cell-derived MSCs [4,36,52]. Generally speaking, these undifferentiated MSCs, possessing the mesenchymal phenotype [4,52], readily re-differentiate from the same lineage or transdifferentiate from other lineages of MSCs into TECs/ECs or other types of stromal cells [54,55]. In addition to the conventional MSCs, particular TECs within the vasculature also gain a mesenchymal phenotype (Vimentin^+^) through EndoMT [114], followed by a loss of cell–cell junctions and downregulation of platelet endothelial adhesion molecule-1 (PECAM-1) [115]. Mesenchymal TECs are subsequently shed from the endothelial lining layer of neoplastic vasculature into peripheral blood and turn into CTECs that may inherit an invasive ability from their parental TECs [52,116]. Another significant contribution of EndoMT is its ability to generate CAFs in the TME [117]. Although CAFs originate from a variety of cells, such as epithelial cells and fibroblasts, dedifferentiation of ECs into MSCs via EndoMT, followed by differentiation of MSCs into CAFs, is the most representative pathway for CAF formation [4,52,117]. CAFs and other stromal cells, vice versa, may dedifferentiate into MSCs [22,118,119], then to TECs/ECs through the transdifferentiation process. Hypoxia induces EndoMT, which participates in the “stromal cell–MSC–EC transition cycle” [53], enabling stromal cells to revert into MSCs and then produce ECs and TECs via either the differentiation or transdifferentiation process. The resultant ECs and TECs are subsequently involved in tumorigenesis and cancer metastasis [52,114]. In particular, EndoMT was found to significantly promote transendothelial extravasation of neoplastic cells during metastasis [4,120]. 

Besides facilitating MSC and CAF formation, EndoMT promotes remodeling of the cytoskeleton in tumor vasculature during cancer cells’ metastatic transendothelial migration, resulting in loss of endothelial adhesion molecules and an increase in vascular permeability. The resultant aberrant vascularity accelerates metastasis through promoting extravasation of cancer cells [120]. Moreover, it has been realized that EndoMT correlates with both reactivation of dormant CD44v6^+^ CSCs [121] and an inflammatory endothelial dysfunction that is associated with tumorigenesis [122]. EndoMT, thereby, largely participates in cancer tumorigenesis, progression, and metastasis [4]. 

### 5.2. Hypoxia Induces Tumor Neovascularization In Vivo and Formation of Cancer Cell-Derived Vasculature In Vitro

In carcinoma cells, hypoxia stress signals could be mediated and delivered by the transcription factor HIF-1, which activates a number of hypoxia response genes and subsequently upregulates angiogenic growth factors. Angiogenesis, vasculogenesis, lymphangiogenesis, and VM constitute the main programs of tumor neovascularization in the hypoxic TME [123]. 

Angiogenesis is primarily promoted by the hypoxia-induced VEGF in the TME, where pre-existing ECs are recruited into the hypoxic area and their proliferation is stimulated [27]. Besides tumor cells, the pro-angiogenic SASP VEGF is also secreted from senescent fibroblasts [124]. Several transcription factors, such as Twist1 and Notch, are involved in the angiogenesis process in the hypoxic TME. For example, following ligand binding, the Notch receptor is cleaved, its intracellular domain translocates into the nucleus and activates a series of transcription events, then participates in angiogenesis [125]. The Notch signaling pathway is a key regulator for both EMT and EndoMT in terms of promoting mesenchymal transformation for both epithelial and endothelial cells [126]. 

Vasculogenesis is mainly driven by the stromal cell-derived factor-1 (SDF-1 or CXCL12), which is a HIF-1-induced chemokine [28]. The interaction between the ligand SDF-1, which is secreted from EPCs, with its receptor C-X-C chemokine receptor type 4 (CXCR4) on neoplastic cells promotes extravasation and development of the pre-metastatic niche [127]. Moreover, EPCs also demonstrate their relevance to enhanced cell migration and invasion, thus confirming the role of vasculogenesis in metastatic niche formation [127,128]. During vasculogenesis, progenitor cells differentiate into ECs which can then be utilized for both heterotypic cell fusion and the transdifferentiation process [28,29]. Hypoxia is therefore a principal inducer for both vasculogenesis and angiogenesis. 

Other than angiogenesis and vasculogenesis, hypoxia-induced EMT significantly impacts the process of VM channel formation. During VM development, several transcription pathways are activated by hypoxia. For instance, the critical EMT-inducing transcription factor Twist1 is driven by hypoxia to translocate from cytoplasm into the nucleus of cancer cells, followed by upregulated expression of the mesenchymal protein, Vimentin and the essential endothelial specific adhesion molecule, VE-cadherin [35]. Induced EMT promotes the generation of mesenchymal CSCs, which subsequently participate in the VE-cadherin-dependent VM channel formation [35,129].

Apart from inducing the transdifferentiation of tumor cells into CSCs and thereupon to ECs during angiogenesis in vivo, hypoxia, which can be created in vitro by reducing oxygen concentration to 1%, was found to enable the cells of several CD31^–^ carcinoma cell lines to transdifferentiate into CD31^+^ TECs and form tubular vessel-like structures in a 3D cell culture [130,131]. Additionally, it is within possibility to imitate an intracellular hypoxic environment in vitro by utilizing the hypoxia-mimetic agent cobalt chloride (CoCl_2_), which promotes the induction of HIF-1α and EMT in several cultured carcinoma cell lines [132,133]. 

### 5.3. Hypoxia-Induced Cell Fusion Participates in Tumor Neovascularization and Cancer Distant Metastasis

Pre-existing ECs in the tumor vasculature and newly generated ECs from either vasculogenesis or transdifferentiation of both CSCs and MSCs fuse with cancer cells to generate either mononuclear synkaryons or multinuclear heterokaryons [57] of TECs, followed by their active participation in neovascularization in the TME. Similarly, neoplastic cells also fuse with MSCs to form hybrids that possess the acquired mesenchymal properties [74], thus increasing their invasion and migratory capability, resulting in enhanced cancer metastasis [68,69]. Heterotypic cell fusion is involved in tumorigenesis and the development of carcinomas [58,61]. The fusion process is significantly promoted by both hypoxia and induced apoptosis via the PtdSer-BAI1 signaling pathway [70] or TNF-α which stimulates the accumulation of intracellular HIF-1 [134]. Cancer cell fusion, mediated by hypoxia-regulated EMT and EndoMT, is a key program in the complex network of tumorigenesis and disease progression. It has been recognized that ECs/TECs in the lymphatic vessel-related lymphangiogenesis and the blood vessel-relevant angiogenesis share the same regulatory pathways within the hypoxic TME [41,123,135]. Hypoxia, the vital inducer of tumor neovascularization and cancer metastasis, activates a series of intracellular events. Such tangled interactions in the TME significantly contribute toward fostering cancer development. 

**Figure 3:**
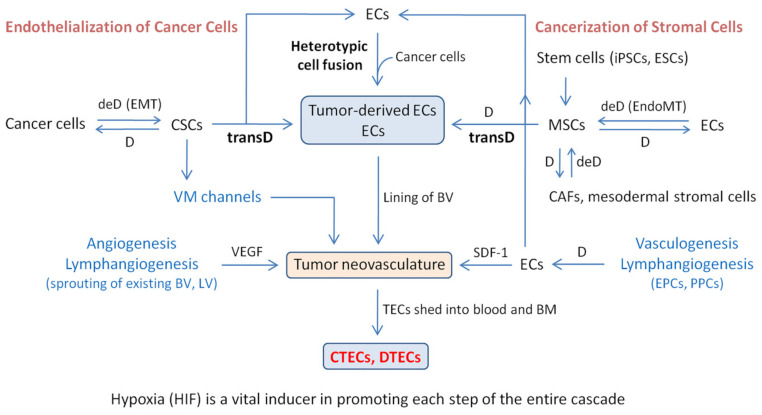
Hypoxia-induced tumor neovascularization cascade generates CTECs in cancer patients. Tumor neovascularization in carcinoma patients is contributed by endothelium-dependent angiogenesis, vasculogenesis, lymphangiogenesis, and endothelium-independent cancer cell-derived VM channels. The lining of blood vessels (BVs) in tumor vasculature is constituted primarily by CD31^+^ tumor-derived ECs. Aneuploid TECs, the endothelialized cancer cells in essence, are derived from both cancerization of stromal cells (such as ECs) and endothelialization of malignant tumor cells, of which the latter consists of the heterotypic cell fusion of cancer cells with ECs and the transdifferentiation (transD) of carcinoma cells via CSCs. In the tumor microenvironment (TME), in addition to participating in channel formation by vasculogenic mimicry (VM), undifferentiated tumorigenic CSCs differentiate (D) into cancer cells, and some of these differentiated cancer cells are able to inversely dedifferentiate (deD) back into mesenchymal CSCs through EMT. Cancerization-relevant MSCs are derived from induced pluripotent stem cells (iPSCs) and embryonic stem cells (ESCs). The dedifferentiation (deD) of ECs and other stromal cells via EndoMT provides an extra origin of MSCs. Subsequent in vivo transD of CSCs and MSCs into TECs/ECs is also observed in vitro. Besides transD, EC-derived MSCs are also able to conversely differentiate (D) into the same endothelial lineage of TECs/ECs in the hypoxic TME. Obtained normal ECs may, in turn, fuse with cancer cells to turn into TECs. Compared to stromal cell cancerization, most TECs actually originate from the endothelialization of neoplastic cells. Vasculogenesis, a process that involves differentiation (D) of bone marrow (BM)-derived epithelial progenitor cells (EPCs) and pericyte progenitor cells (PPCs) into ECs, is an additional source of ECs for heterotypic cell fusion. BVs and lymphatic vessels (LVs) share the same pathways to generate ECs and TECs. CTECs and DTECs are aneuploid TECs that are respectively shed into the peripheral blood and BM. During the formation of TECs and CTECs, both EMT and EndoMT, which have identical transcription pathways and the same core set of EMT-inducing transcription factors (EMT-TFs: Twist1, Snail, Slug, ZEB1, ZEB2, and Notch), are critically involved in the formation processes. In the illustrated CTEC formation cascade, hypoxia is a vital inducer for every step, EMT and EndoMT are the crucial hubs, CSCs and MSCs are the central nodes, TECs are the essential contributors, and mobile CTECs and DTECs are the critical versatile players. D, differentiation; deD, dedifferentiation; transD, transdifferentiation.

As depicted in Figure 3, a schematic framework illustrates how CTECs are formed along the cascade of hypoxia-driven neovascularization. TECs are contributed by both “endothelialization of cancer cells” and “cancerization of stromal cells”. During the endothelialization process, undifferentiated tumorigenic CSCs differentiate (D) into cancer cells or transdifferentiate (transD) into TECs. In the TME, the differentiation process of CSCs is bidirectional, meaning that differentiated cancer cells are able to conversely dedifferentiate (deD) into mesenchymal CSCs via EMT, followed by subsequent transD into TECs. The “endothelialization of cancer cells”, composed of CSC transD and heterogenic cell fusion, is likely the primary origin of TECs. In the process of “stromal cell cancerization”, induced pluripotent stem cells (iPSCs) and embryonic stem cells (ESCs) give rise to MSCs. The dedifferentiation (deD) of stromal ECs and other lineages of stromal cells (such as CAFs and mesodermal stromal cells) via EndoMT constitutes an additional origin of MSCs. BM- and EC-derived MSCs may respectively transdifferentiate and differentiate into TECs/ECs based on their derivation lineages. Both EMT and EndoMT share the same signaling pathways. In the depicted cascade of CTEC formation, hypoxia acts as a pivotal trigger, EMT and EndoMT work as the crucial hubs, CSCs and MSCs are the central nodes, ECs and TECs in lymphatic and blood vessels are the essential contributors, and CTECs are the important players in tumor neovascularization and cancer metastasis. Together with Jagged1/Notch/KLF4, hypoxia-induced EMT-TFs are the prime regulators for the entire processes [7,108].

## 6. Clinical Significance and the Novel Therapeutic Target Potential of Metastatic CTECs 

### 6.1. TECs and CTECs in Hematogenous and Lymphogenous Cancer Metastases

Aneuploid TECs, which express the endothelial marker CD31 and harbor dual properties of both cancerous malignancy as well as endothelial vascularization ability, are the malignant cancer cells in essence. These cells are often referred to as a “wolf in sheep’s clothing” [59]. Given that TECs account for as high as 70% of the ECs in tumor vasculature, aneuploid TECs exhibit a significant impact on tumorigenesis, progression, invasion, and metastases (both hematogenous and lymphogenous). 

**Figure 4:**
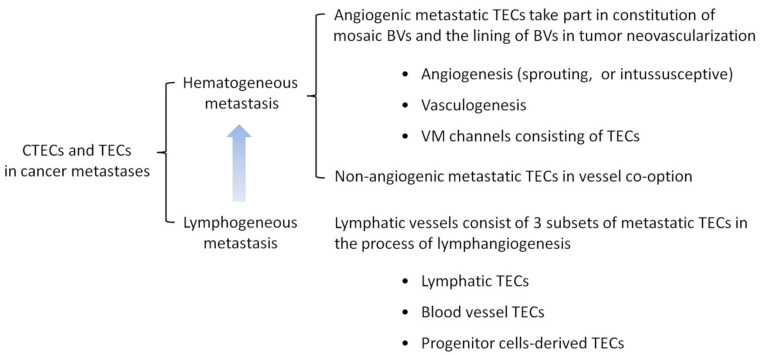
Participation of CTECs and TECs in cancer metastases. TECs constitute a significant proportion of endothelial composition in lymphatic and blood vessels (BVs) of tumor vasculature. Following shedding into peripheral circulation or lymph flow, TECs turn into CTECs. CTECs actively participate in hematogenous and lymphogenous cancer metastases. Lymphogenous metastatic cancer cells and CTECs in the lymph eventually converge into the peripheral blood of hematogenous distant metastasis.

Aside from sharing the TECs’ properties, CTECs and DTECs acquire additional migration ability, which may provide these circulating rare cells unique capabilities along the metastasis cascade including intravasation, circulating, targeting the specific organ, extravasation, seeding, neovascularization, tumor progression, and susceptibility or resistance to cancer therapy. Similar to CTCs, CTECs could be phenotypically and karyotypically classified into diverse subtypes based on cell size, quantified aneuploidy of chromosomes, and TM expression [14]. Different subtypes of CTECs, such as TM-positive or null CTECs, may specifically possess distinct clinical significance. Apart from CTCs and the metastatic carcinoma cells in malignant lesions, the proposed participation of TECs and CTECs in hematogenous and lymphogenous metastases is illustrated in Figure 4. 

### 6.2. Co-Detection of CTC and CTEC Subtypes to Predict and Evaluate Therapeutic Efficacy of Anti-Angiogenic Regimens

Bevacizumab (Avastin) is the only approved anti-angiogenic monoclonal antibody for the first-line treatment of metastatic lung, breast, and colorectal cancers. Bevacizumab targets cancer cells’ secreted VEGF-A, disrupts its binding to the VEGF receptor on ECs or TECs, and subsequently abolishes VEGF’s angiogenic activity [136]. A combination regimen of chemotherapy with bevacizumab showed increased response and improved progression-free survival (PFS) in cancer patients compared with chemotherapy alone. However, a substantial portion of cancers have a pre-existing intrinsic or acquired resistance to bevacizumab, significantly undermining its clinical application [137]. The establishment of reliable biomarkers used to select eligible patients and evaluate the anti-angiogenic therapeutic efficacy in real time is an insistent clinical demand.

Conventional quantification of circulating endothelial cells (CECs) to evaluate bevacizumab efficacy in patients with a variety of cancers has been described elsewhere. Among the published efforts by others, very few studies attempted to co-evaluate the clinical utility of CECs and CTCs in patients during anti-angiogenic treatment [138]. Moreover, compared to the cancer cell-derived aneuploid CTECs, ordinary CECs may have less malignant cancer-related specificity as well as clinical relevance to anti-angiogenic efficacy in carcinoma patients. The *in situ* phenotypic and karyotypic characterization technology, iFISH, has been developed (Figure 2) [11,14,76], making it possible to co-detect aneuploid CTEC and CTC subtypes in patients subjected to therapy combining anti-angiogenic bevacizumab with chemotherapy (such as paclitaxel or platinum-based chemo agents), immunotherapy (such as immune checkpoint inhibitors), or anti-HER2 (such as trastuzumab/Herceptin) or anti-EGFR (such as gefitinib/Iressa, erlotinib/Tarceva, and tyrosine kinase inhibitors TKIs) targeted therapy at designated time intervals. Given that EMT and EndoMT are substantially involved in tumor neovascularization and that the mesenchymal molecule Vimentin is regarded as an independent prognosticator for poor survival [139], it is ideal to integrate Vimentin and/or EpCAM into the iFISH co-detection platform [11,12]. Extensive clinical studies performed with such co-detection and comprehensive characterization of CTCs and CTECs will provide valuable perceptions regarding how to more appropriately select an eligible cohort of patients without intrinsic resistance to the treatment, to monitor therapeutic efficacy in real time, to precisely identify single cell-based specific subtype of CTCs or CTECs either sensitive or resistant to therapy, and to provide an insightful comprehension concerning how CTECs and CTCs reciprocally coordinate in metastatic cancer patients treated with anti-angiogenic or combination therapy.

### 6.3. CTEC: An Emerging Therapeutic Target in Motion

A great endeavor has been taken to try to enhance the efficacy of cancer therapies for decades. However, current therapeutic efficacy has not yet met clinical expectations. Several biological determinants induce therapeutic drug resistance, including tumor heterogeneity, undruggable genome, immune system, and the TME [140,141]. Accumulated evidence has indicated that different types of carcinoma cells and TECs may have inherent resistance to treatment regimens. For instance, in addition to cancer cells, HCC-derived TECs were resistant to chemotherapy [142]. Considering the high intratumor heterogeneity among neoplastic cells of the same type of cancer, and the high intertumor heterogeneity among diverse types of carcinomas in different patients, an early therapeutic intervention to effectively restrict cancer cells from metastasizing, rather than trying to suppress or eliminate all the heterogeneous neoplastic cells in the malignant lesion, is a more sensible choice for clinical management. 

CTCs have been recognized as a prognostic marker for patients with metastatic breast, colorectal, and prostate cancers. CTCs are formally assessed as a surrogate biomarker for overall survival in several ongoing phase III clinical trials [143] and routinely quantified in many pre-clinical studies. In contrast to the conventional CTC enumeration method, which can create a false negative detection hurdle due to restriction to the EpCAM and CK double-positive CTCs, co-detection and *in situ* phenotypic as well as karyotypic characterization of circulating rare cells performed by SE-iFISH [11,14,76] have successfully pinpointed the specific subtype of CTCs and/or CTECs with susceptibility or resistance to the therapeutic agents regardless of CK or EpCAM expression. In one pre-clinical study performed with SE-iFISH, it was revealed that, in the stomach-to-lung metastatic PDX (mPDX) tumor mouse model, the enriched specific subtype of multiploid (≥ pentasomy 8) CK18^+^ non-hematologic cells in circulation was precisely identified by iFISH to possess a chemoresistance to cisplatin [84]. Clinical studies performed on gastric cancer patients indicated that non-hematologic circulating rare cells with trisomy 8 showed an intrinsic resistance to cisplatin, whereas multiploid (≥ pentasomy 8) cells demonstrated an acquired resistance [144]. Additional clinical studies showed that HER2^+^ aneuploid circulating rare cells were related to gastric cancer patients’ development of therapeutic resistance [81]. EpCAM^+^ aneuploid rare cells in peripheral blood and BM significantly correlated with systemic metastasis in breast cancer patients [82]. Moreover, Vimentin^+^ lung cancer rare cells at baseline were relevant to hepatic metastasis [145]. Furthermore, postsurgical small EpCAM^+^ multiploid rare cells in circulation were found to correlate with HCC patients’ postoperative recurrence [83]. Recently, following the positive detection of multiploid EpCAM^+^ CTECs in locally advanced breast cancer patients receiving neoadjuvant chemotherapy [101], multiploid and triploid PD-L1^+^ CTECs were reported to respectively show intrinsic and acquired resistance to the immunotherapeutic agent nivolumab (Opdivo) in NSCLC patients [12]. 

CTECs have been positively detected by us in thousands of clinical specimens from patients with a variety of cancers including lung, breast, colorectal, prostate, melanoma, pancreatic, gastric, ovarian, renal cell, bladder, esophageal, sarcoma, and hepatocellular carcinomas. Aneuploid CTCs and CTECs, plus DTCs and DTECs, comprise cellular circulating tumor biomarkers in carcinoma patients. These subcategorized circulating rare cells may possess an interplay in circulation, and subsequently promote disease progression. TECs’ contribution to tumor progression was previously illustrated [9]. CTECs, integrating multiple properties of epithelium, endothelium, mesenchyme, aneuploidy, malignancy, and motility, have been recognized as a unique category of malignant cells in motion. CTECs are expected to play an important role in tumor initiation, progression, metastasis, and neovascularization. Compared to CTCs, CTECs appear to be more relevant to disease progression in cancer patients [12]. Innovative attempts, beyond conventional anti-angiogenic efforts, to therapeutically target EndoMT and aneuploidy, which vitally impact aneuploid CTECs have recently gained close attention in the field [114,146]. As a potentially distinctive, novel, and mobile therapeutic target, the disruption or elimination of CTECs in cancer patients may effectively obstruct cancer metastasis. Further in-depth genomic and proteomic comprehensive investigation of CTECs and their subtypes will facilitate the discovery of molecular targets for anticancer therapy, unraveling molecular mechanisms in terms of cancer metastasis and susceptibility or resistance to the current, being developed, or to be developed anticancer agents.

## 7. Summary

Aneuploid TECs, accounting for a majority of ECs that constitute the tumor vasculature lining, are a unique category of malignant cancer cells inducibly endothelialized to express the endothelial phenotype in the hypoxic TME. Hypoxia-induced generation of TECs and CTECs in cancer patients is contributed by the cancerization of stromal ECs and the predominant endothelialization of carcinoma cells. The latter consists of cancer cell–EC fusion and malignant carcinoma cells’ transdifferentiation. During the formation of TECs and CTECs, hypoxia-triggered EMT and EndoMT are vital in the formation processes. Aneuploid CD31^+^ CTECs and CD31^–^ CTCs compose a pair of cellular circulating tumor biomarkers that may have an active cross-talk and interplay in circulation, thus promoting lymphogenous and hematogenous cancer metastasis as well as disease progression. The technology for co-detection and comprehensive characterization of the diverse subtypes of aneuploid CTCs and CTECs has been developed, benefitting current and future cancer research, stratification, and personalized clinical management for cancer patients. CTECs, harboring the varying properties of malignancy, vascularization, and motility, are the significant versatile cellular players in tumor neovascularization and cancer metastasis. CTECs may serve as a unique novel target en route toward developing a therapeutic intervention strategy for metastasis blockade cancer therapy. 

## Abbreviations

CSC, cancer stem cell; EC, endothelial cell; EMT, epithelial-to-mesenchymal transition; EndoMT, endothelial-to-mesenchymal transition; MSC, mesenchymal stromal/stem cell; SASP, senescence-associated secretory phenotype; TM, tumor marker; TME, tumor microenvironment; transD, transdifferentiation.
